# Silencing trust: confidence and familiarity in re-engineering knowledge infrastructures

**DOI:** 10.1007/s11019-020-09957-0

**Published:** 2020-05-28

**Authors:** Rune Nydal, Gaymon Bennett, Martin Kuiper, Astrid Lægreid

**Affiliations:** 1 Programme for Applied Ethics, Department of Philosophy and Religious Studies, Norwegian University of Science and Technology, NO- 7491 Trondheim, Norway; 2 School of Historical, Philosophical, and Religious Studies, Arizona State University, Tempe, AZ 85287-4302 USA; 3 Department of Biology, Norwegian University of Science and Technology, NO-7491 Trondheim, Norway; 4 Department of Clinical and Molecular Medicine, Norwegian University of Science and Technology, NO-7491 Trondheim, Norway

**Keywords:** Trust, Familiarity, Moral habitus, Knowledge bases, Knowledge infrastructures, Systems biology, Domesticating futures

## Abstract

In this paper, we tell the story of efforts currently underway, on diverse fronts, to build digital knowledge repositories (‘knowledge-bases’) to support research in the life sciences. If successful, knowledge bases will be part of a new knowledge infrastructure—capable of facilitating ever-more comprehensive, computational models of biological systems. Such an infrastructure would, however, represent a sea-change in the technological management and manipulation of complex data, inducing a generational shift in how questions are asked and answered and results published and circulated. Integrating such knowledge bases into the daily workflow of the lab thus destabilizes a number of well-established habits which biologists rely on to ensure the quality of the knowledge they produce, evaluate, communicate and exploit. As the story we tell here shows, such destabilization introduces a situation of unfamiliarity, one that carries with it epistemic risks. It should elicit—to use Niklas Luhmann’s terms—the question of trust: a shared recognition that the reliability of research practices is being risked, but that such a risk is worth taking in view of what may be gained. And yet, the problem of trust is being unexpectedly silenced. How that silencing has come about, why it matters, and what might yet be done forms the heart of this paper.

## Introduction: knowledge infrastructures and the problem of trust

Efforts are currently underway, on diverse fronts, to build digital knowledge repositories to support research in the life sciences.^1^ To the extent that they are successfully constructed, adopted, and managed, such knowledge bases will help scientists find, share and mobilize the vast volume of knowledge being produced at high speed in the life sciences. Sharing knowledge in a more standardized and automated fashion will also allow them to analyze large datasets and develop ever-more comprehensive, computational models of biological systems. Knowledge bases will however, as this paper argues, constitute a generational shift not only in technical efficiency and scope, but in the manner in which basic scientific questions get asked and answered. Knowledge bases therefore have a profound impact on how biologists relate to the collective knowledge produced in their field, inducing fundamental changes in how biologists capture, circulate, and make use of the collective past achievements of their peers and how they contribute to new discoveries and innovations.

At the heart of these transformations is a historic shift that can be usefully summarized as a move from the published paper as the prime instrument of knowledge management, to the digital object. Since the advent of modern biology, biologists have been trained to communicate their research findings through a common body of literature. The formulation, conduct and defense of their experimental work relies heavily on this literature and on a shared set of customs for finding, reading and evaluating other biologists’ knowledge claims, as well as narrating, reporting, and circulating their own. Knowledge bases disrupt and reorient these learned practices. They do so by extracting the knowledge embedded in published papers and representing that knowledge in standardized, logically structured, databases available in a machine-readable form. Knowledge bases enable researchers in the life sciences to get an overview of what is known—but also ask and answer questions at a scale of complexity and degree of automation beyond what would be possible using published papers or even standard databases. Knowledge bases thus promise to take care of what is already known and open up new research trajectories across the life sciences. The task of realizing this potential, however, is more than just technical, it reorients biological practice. Biologists now have to familiarize themselves with digital knowledge bases, including the institutions, processes and computational expertise involved in the work of building and maintaining them.

The significance of this reorientation can be helpfully clarified by viewing knowledge *bases* in the context of the knowledge *infrastructures* of which they are being made to form an integral part (see Edwards et al. 2013). Knowledge infrastructures, to quote Edwards et al., are “robust internetworks of people, artefacts, and institutions which generate, share, and maintain specific knowledge about the human and natural worlds” (2013, p. 23). Knowledge infrastructures are at the same time social and material. They constitute the ensemble of interactions needed to ensure the production and reproduction of scientific knowledge. The term ‘robust’ is key. What makes a knowledge infrastructure robust is that it allows for the stable production and reproduction of knowledge with enough quality and fidelity that the people using it can get work done in a manner that is more or less unreflective and unreflexive. Robust knowledge infrastructures, to borrow a classical term from the social sciences, can be said to form part of the ‘moral habitus’ of the biosciences (Mauss 2000). Robust infrastructures are familiar: the people who use them don’t have to think about them too.

The introduction of knowledge bases impacts such familiar taken-for-granted practices. It forms part of a now decades-long disruption of robust infrastructures in the life sciences brought about by the introduction of computers into nearly every aspect of laboratory life. That disruption “destabilizes” robust infrastructures inasfar as it puts in question the tools and processes used to publish, access, circulate and exploit knowledge. It puts them in question in the literal sense of raising them from the background of the daily habitus to something that must be actively considered. Knowledge bases do this by introducing a computational proxy for the published paper, effectively displacing the published paper as the primary instrument in the circulation, management, and use of knowledge. Knowledge bases thus require researchers, at least in the short run, to rethink and retool the knowledge infrastructure they may have previously taken for granted. At the same time, in order for knowledge bases to become part of a new robust knowledge infrastructure, the destabilizations they introduced will need to be re-stabilized. Knowledge bases will need to become, in other words, part of a new moral habitus in biological research, one in which they have been incorporated into a, yet again, taken for granted way of doing work.

### The question of trust

This situation would appear to be one that requires—in the sociologist Niklas Luhmann’s technical sense of the term—trust. Trust, Luhmann proposes, is required in any situation where a bad outcome of the choices you are making would cause you regret having made those choices: “a situation where the possible damage may be greater than the advantage you seek” (Luhmann 2000, p. 98). Trust, importantly, can be thought of as a mode of approaching a situation which has become *unfamiliar* in the sense that normal courses of action can no longer be taken for granted. A mode of trust is needed in situations in which unfamiliarity makes you aware of the stakes involved and in which awareness of those stakes activates a conscious sense of potential risk. You can be said to be operating in a mode of trust if you are aware of a risk that you nevertheless are willing to take in view of what may be gained—the hoped-for futures you aim to realize.

A question at the heart of this paper—and of the ongoing work of which this paper forms one part—is this: does the introduction of new digital knowledge repositories and the re-engineering of knowledge infrastructure of the life sciences this entails, require trust or not? And if so, under which conditions and to what ends? The question of trust is important because how one answers it makes a difference for what one considers to be the appropriate attitude towards the possible risks involved. Importantly for our analysis is the distinction Luhmann makes between *trust* and *confidence*. Trust *does not* mean having a ‘high degree of confidence’, but rather a conscious response to an acknowledged situation of risk. To the extent that the risks involved call for trust, researchers might be expected to embrace and operate in a reflective and reflexive work mode, open to possible alternatives and attentive to reasons for resistance and non-compliance, even as they move forward. If the introduction of knowledge bases warrants a mode of confidence, however, then building and adopting them can simply be cast as the natural next step for the life sciences. This step may require hard work, but it can be cast as work researchers *should feel committed to do*, at least do their fair share for the scientific common good. Trust, in short, names a mode that entails recognizing risk and attending to possible resistance. Confidence entails complying with dominant norms by moving forward with the assurance that risks have been adequately dealt with.

At stake is a question of what a responsible work mode is in this situation. To the extent that the work requires a *mode of trust*, yet is carried out as if it is principally a *matter of confidence*, the biological community risks taking unexamined epistemic and moral risks. And yet, if a mode of confidence is called for, working in a *mode of trust* may invite epistemic blockages and higher opportunity costs than warranted. Crucially, in practice this is rarely, if ever, an either-or situation. The question is thus one of the appropriate ratios of these modes and the persistently difficult question of what forms of practice ought to embody those ratios. Moreover, the answer to these questions will be different in situations where researchers are being asked to help build knowledge bases and situations—often overlapping—where researchers are being asked to become consumers and users of knowledge bases built by others.

### The question of modes

This paper takes the view that the question of trust and what it requires in terms of practice *should be* handled as an open one. We suspect that the current situation requires a combination of trust and confidence. This implies that researchers should actively consider whether, and under what conditions, a mode of trust or confidence is appropriate and productive. How and under which conditions that difference should be activated is a question for another paper. In this paper we focus instead on i) why it is reasonable to ask whether there is an epistemic risk even if the situation feels familiar, ii) how the silencing of trust may take place in the ordinary work to build and adopt knowledge bases, and iii) what is at stake in closing the question of trust. In the sections that follow, we aim to establish our argument by means of telling a story in three parts. The *first part* describes how the knowledge infrastructure in biology was destabilized by the introduction of technologies for systems-wide genomics analyses two decades ago. In our account, the invention of knowledge bases was primarily meant to address that destabilization. This part of the story aims to show how this work of building knowledge bases can be understood as part of a radical re-engineering of the knowledge infrastructures of the life sciences.

Despite the radical nature of the shift under way, the work of building and adopting knowledge bases is today being undertaken in a mode of confidence rather than trust. This raises the question whether trust is being unduly silenced and how that may happen. This may happen gradually, as we suggest in *part two*, as the newly evolving digital knowledge configurations grow familiar—in the sense that researchers have become accustomed to using them (Luhmann 2000). This appeal to familiarity and confidence demonstrates a general feature described by Bruno Latour (1993): ordinary research tends to hide the transformations it enacts. In our account, this happens in a process we describe as a gradual ‘domestication of the future’. The work of building and adopting knowledge bases is guided by a vision of the future. This desired future gradually becomes a normalized confident position from which one feels at home and the tasks of the present are oriented, undertaken and reassured as the natural next step for science. This second part of the story aims to show that such domestication of the future may well have covered over the need for a more deliberate and considered approach to the relation of trust and confidence in the building and adopting of knowledge bases.

In the *third part* of the paper we reflect on the costs of silencing trust. Proposing that risk is necessary for any scientific undertaking worthy of the name, we conclude by suggesting that unless the silencing of trust is lifted, researchers risks not to be able to achieve the futures they imagine and hope for.

## Part I: When the paper became a problem: a story of breakdown

Our analysis is most immediately informed by a contemporary initiative that we have been part of since 2013: the Gene Regulation Consortium (GRECO).^2^ GRECO targets the specific domain of molecular knowledge relevant for the cellular process of gene regulation. The aim is to build a common knowledge management framework to support researchers in this field where knowledge bases form a key part. The project is both technically and scientifically difficult. It involves, in the first place, the long and difficult process of developing reliable technologies needed for the automated extraction of knowledge about gene regulation found in published papers and developing digital standards needed to represent that knowledge in a manner consistent with existing data tools. In the second place, all of that will need to be done in a way consistent with generally accepted theoretical understandings of the dynamic mechanisms of gene regulation^3^ and thereby supportive of the wide range of research interests and needs of the biological community.

To the extent all this is successful, knowledge bases will have introduced a major shift in the daily life of the gene regulation researcher. The existing knowledge infrastructures, revolving around the published paper, consist of internetworks of people, artifacts, and institutions like publishing houses, editors, librarians, libraries, networks of libraries, peer review, the Vancouver declaration, paper genres, impact-factors, inclusion and exclusion criteria, journals, and peer-review systems, all knit together in an economy of public and proprietary knowledge. The infrastructures that revolve around the digital object will require recalibrations across all of these: a new regime of knowledge curators, new ontologies for biological data, procedures for data-mining, metrics for quality control, new controlled vocabularies for data annotation and management, professional credit for nano-publications, new requirements for validating and sharing information and the like.

What is remarkable here, to emphazise the radical nature of this shift, is not the need for epistemic adjustment per se. Such processes are an integral part of ordinary experimental research. Biologist regularly find themselves in a situation where adjustment and refinement of their infrastructure is called for. As Hans-Jorg Rheinberger put it, experimentalists create ‘experimental systems’ that are “systems of manipulation designed to give unknown answers to questions that the experimenters themselves are not yet able clearly to ask”. Such setups are, as [the biologist Francois] Jacob once put it, ‘machines for making the future’. They are not simply experimental devices that generate answers; experimental systems are vehicles for materializing questions” (Rheinberger 1997, p.28). Ordinary experimental work involves work of recalibrating one’s experimental systems in order to *materialize* questions one otherwise would not be able to ask—and importantly does not really know how to otherwise articulate. Knowledge bases, however, represent not simply a matter of ordinary adjustment, but a fundamental recalibration of what it means to ask and answer good scientific questions, how to go about that, and what tools will be required. They represent adjustments at the level of the knowledge infrastructure itself and not only at the level of the experimental systems which otherwise rely on it.

### From the microarray to the knowledge base

All of this can be illustrated by a story, which lies behind the efforts to create GRECO. Many researchers in the late 1990s came to realize that the published paper was increasingly becoming an obstacle for efficient and manageable circulation of knowledge in biology. This became especially pressing at the turn of the century for so-called post-genomic researchers building experimental platforms designed to exploit the new lines of inquiry being opened up by the genome sequencing projects of the previous decade. One of the most promising of these technologies was called the DNA microarray—a small chip that would allow researchers to measure the expression levels of large numbers of protein-encoding regions of DNA simultaneously, offering the potential of a genome-wide view on gene-expression.^4^ In the late 1990s, one of the authors of this present paper spearheaded an initiative to establish microarray platform technology in Trondheim, Norway, to be used to obtain such a systems-level view. In order to draw scientifically meaningful inferences about such experiments, however, it was clear that the researchers would need not only the microarray technologies themselves, but also a means to take into account current knowledge about thousands of genes and their interrelations. This turned out to be a fundamental limitation, one calling for the creation of knowledge bases.

The research group in Trondheim was investigating the molecular mechanisms involved in gastric cancer (stomach cancer). They had acquired a feeling for the way in which hormones play a role in the regulation of gastric acid secretion and possibly thereby, gastric cancer. The hormone gastrin had become one of their favourites, so to speak, an object of investigation pivotal to their understanding and experimental work. They knew its patterns of behavior quite well—and suspected that it played a key role in stomach cancer. They could spot gastrin’s unusual action but were painfully aware of the long journey of experimental work they would have to undertake to establish the molecular basis for how gastrin worked in cancer. On one level, few people in the world could evaluate gastrin actions and responses better than they could. And yet, for all this, they only had intimate knowledge of a handful of the (possibly large numbers of) genes and proteins involved. They did not have a system’s level view of the problem. It followed that their models of gastric cancer were necessarily limited in complexity.

Microarray technologies opened a new possible way forward. They carried expectations of a fundamental shift in capacity—a potential game-changer with regard to understanding the causal mechanisms underlying stomach cancer and the role of gastrin therein. With the microarray they could record the entire genomic response of a cell stimulated by gastrin, rather than the one-gene-at-a-time approach they had been stuck with previously. The prospect, however, brought new kinds of uncertainty. The use of microarrays constituted unfamiliar scientific terrain, raising questions for how to establish scientific evidence for the causal significance of the gene expression levels microarrays would allow them record. Not only did the technological platform have to be built from scratch, digital analytical tools had to be built capable of managing the information about the thousands of genes being interrogated. Importantly, such tools had to account for what was currently known in the field about these genes. But how could one get reliable information about what is known about all relevant genes in a manner that would facilitate comparative analysis and potentially a systems-level understanding of the problem?

By the late 1990s, a number of efforts were underway to coordinate and standardise the annotation, collection and representation of current knowledge about genes in a digital form. LocusLink and the Gene Ontology Consortium were two prominent initiatives (Maglott et al 2000; Ashburner et. al. 2000). These resources, however, were not yet developed enough to provide the researchers in Trondheim with the comparative, systems-level information they needed to make sense of the data they were producing about the genes involved in the growth and regulation of gastric cancer. Limited by the relative immaturity of these tools, they did what researchers do: they looked through the literature.

The research team needed to collect up-to-date information about the roughly 500 genes whose expression levels they were observing in their experiments. The trouble was, every gene had its own *individual* silo of fast-growing literature. To make matters worse, for most of the genes they were investigating, they did not have first-hand knowledge of gene function—they didn’t know what the genes were thought to do. The best they could do under these circumstances was to pick one or two literature abstracts on each gene from a stack of search results provided by Medline, and trust their accumulated general expertise in making sense of the reports. Sitting around a table in January 1999, two senior researchers described one gene at a time, glancing at abstracts and consulting the other’s opinion. They were clearly uneasy about the scientific limitations of the ad-hoc knowledge repository they were creating for themselves. They even joked about how fragile their research must look like from the point of view of the humanist in their lab. And yet, they saw no better solution.

It was at this point that the researchers began to cast the published paper—the historically primary medium and anchor point for the circulation of knowledge—as a problem. They were asking questions of the published paper that the format was simply not designed to help answer—even with the help of the search functions in Medline. Familiar ways of accessing and exploiting relevant knowledge constituted an obstacle to taking full advantage of the system’s level view made available by the microarray technology.

In the late 1990s, microarrays can be said to have made manifest a powerful new vision of the future of biological research, one whose realization required new infrastructures and thus the taking of new epistemic risks, but whose far-reaching potentials made those risks clearly worth taking. Moreover, since microarray technology was one of the first tools of genome-wide analysis to be established, it was taken to be essential to actualizing a vision for system-wide investigations of the organism more broadly, an approach that would open up powerful new biotechnological innovations. By 1998 microarrays (gene chips) had found their way into US President Bill Clinton’s “State-of-the-Union” speech with the following promising statement; “gene chips will offer a road map for prevention of illness throughout a lifetime” (Phimister 1998). It was presented as the “Array of Possibilities” (Marshall & Hodgsonn 1998) or the “Array of Hope” (in Nature Genetics special issue “The Chipping forecast”, Lander 1999), that would accommodate "The Next Revolution in Molecular Biology" (Gwynne & Page 1999).

Such a grand vision of the future was, however, predicated on the possibility of a new lifeworld for researchers, one in which the imagined microarray capacities could be rendered stable, productive, and robust. That is to say, that vision was predicated on the creation of new knowledge infrastructures, including the machinery of knowledge management such data-centric infrastructures clearly required. The vision seemed self-evident, the road to realizing that vision far less so.

For their part, the researchers in Trondheim turned to a colleague in the computational sciences for help. They knew they needed to find ways to use computers to extract and represent the knowledge about the genes they found in the literature. They needed a knowledge base, and thus found themselves dependent on a different mode of expertise (i.e. computer science) to take the next step in their work. The computer scientists had tools at hand, developed for similar problems in otherwise unrelated contexts, that could potentially be adapted and adopted for biology. Known as tools for ‘Data Mining’ and ‘Knowledge Discovery’, these technologies had initially been developed to extract patterns of consumer behavior from large complex data sets. Together, the biologists and the computer scientist undertook to extract knowledge of gene function from the titles and abstracts of ten million Medline publications.

Their methods were scientifically crude: they searched for gene names and functional terms, assuming the article reported a gene–gene relation if two gene names were mentioned. However scientifically unsatisfying, their method appeared to be as close one could get at the time to the kind of broad collection of knowledge claims they needed, to conduct comparative analysis. As they it put it in their publication, it was at least a first step toward a “literature-wide as well as genome-wide view of the current knowledge about human genes” (Jenssen et al. 2001).

In a strangely reflexive twist, the language of the Jenssen et al. (2001) paper bears witness to a moment in history when the published paper first began to appear as a problem for biology. It was the kind of problem that induces what James Moor (2005) described as ‘conceptual muddles’: we struggle to find adequate modes of reasoning for situations created by the introduction of significant new technologies, even while we rely on those technologies to deliver what we take to be the best possible response to the situation we find ourselves in. Current knowledge, as they described it in the paper, had become something that one was forced to ‘recycle’: the knowledge contained in a given paper needed to be extracted not only in view of the experimental system in relation to which the paper had been written, but it also needed to be repositioned as one element in a complex field of relations well beyond the silo of its original context. Crude computational methods were used in order to extract or “rediscover” knowledge from a sea of literature—a task that was simply unworkable at the level of the human researcher given the constraints of time and talent. The “background knowledge” needed was beyond the scope of “manual interpretation”. Knowledge, although carefully taken care of by a number of different communities, had to be rediscovered and represented in a standardised computable format adjusted to the need of the research program at the user end.

The story illustrates how the microarray called for the creation of a technology of knowledge management that effectively flattened and thinned out researchers’ relation to prior knowledge in the field. The rich thick intimate knowledge of gastrin, for example, that the researchers had developed in their previous work stands in sharp contrast to the thin crude method of harvesting knowledge by scanning abstracts with a computer looking for key words. Yet efforts like this offered the best available way forward. Someone somewhere may have intimate knowledge of any one of the 500 genes allegedly involved in gastrin-related stomach cancer, and they may have even published on those genes. But that did not help the scientist in Trondheim. For all practical purposes, current knowledge of genes and their behaviour might not have existed if it could not be extracted, turned into a digital object, and made a part of a standardized knowledge base.

With the microarray (alongside other post-genomic technologies), paper-centric knowledge infrastructures could no longer be taken as adequate to the future of biology and biotechnology. Such futures required infrastructures anchored in and animated by knowledge bases. Post-genomic technologies were disruptive and destabilizing inasmuch as they allowed biologists to imagine a radically different future. *That imagined future was taken to be one worth building, even at the cost of a radical reworking of existing experimental practices*. The risks involved called for trust.

In this situation an important shift began to take place, one which is crucial to our story and to this paper. Under these “radical” conditions of reimagination, in which a new vision of scientific and technical capacity became predominant, researchers were put into a position where they started engaging the present from the point of view of the imagined and desired future, a future in which knowledge bases had been built and put to use as a normal part of research practice. Such a domestication of the future, as our story aims to show, needs to be taken into account in evaluating the present situation in which the work of building and adopting knowledge bases is experienced and carried out as if it is principally a matter of confidence. The situation may still call for trust even if it feels familiar.

In our account, a strange if largely unacknowledged inversion took place over the years that followed. Rather than the technological and scientific future appearing as uncertain in relation to the present, the present began to be seen as “caused”, that is to say, directed by and toward, the imagined future. The hoped-for future began as a project full of risks worth taking but came to govern the expectations and appetites of the present. In this way the project may gradually have begun to lose any character of riskiness at all simply because one has become so used to it. The reason for this, as we argue in the next section, is that the imagined future, which might otherwise be taken as unknown and therefore unfamiliar, has been ‘domesticated’, in the literal sense that researchers have begun to feel at home with it. Such domestications are likely to happen in all transitions; it is part of the dynamics of ordinary research. In the case of microarrays, and the host of systems-wide technologies they formed a part of, a future infrastructure for systems biology, however unknown in its technical details, began to seem not only desirable but inevitable. The unfamiliar was made familiar in as far as present day work began to be governed in view of it. To that degree, the work of building knowledge bases and making them part of the taken for granted moral habitus of biology is no longer experienced as an epistemic shift and thus a risk. As this happens, the question of trust may be silenced in unproductive and irresponsible ways.

## Part II: Familiarity and domestication: the politics of reassurance

The domestication of the imagined future in which knowledge bases are up and running has an important scientific dimension to it. The imagined future, in which the infrastructure has been normalized, recalibrates researcher’s sense of the kinds of scientific questions they ought to be asking and the kinds of technical applications they should be pursuing. This becomes more than just the positing of a hoped-for goal. Rather, it constitutes a process of extrapolation from the destabilized situation of today, to the imagined stable situation of tomorrow, an extrapolation that has determinative effects for what can and should be taken for granted today. That stable future then forms another loop, retroactively giving further direction to the work of constituting the present. In this way—and this is the key point—the unsettled dimensions of the moment that we are living in can be rendered not only tolerable but invisible.

The process itself is so normal it almost goes unnoticed. Consider the GRECO project. GRECO is animated by and oriented to a world in which an imagined web of knowledge bases has matured and in which causal information about gene regulation is integrated into the full range of knowledge management tools used in experimental research. On that view, the choice to pursue knowledge bases today seems not only scientifically reasonable, but inevitable. Yet such inevitability could not have been taken for granted even a decade ago. As the researchers in Trondheim set out to analyze microarray data as recently as 2010, the knowledge they needed was not available in existing knowledge bases. Annotations for only about 300 of the expected 1500–2000 transcription factors could be found in knowledge bases like Gene Ontology—one of the best available tools. Once again, as they had a decade earlier, they turned to the literature. But now things were different. In 2010 they had higher expectations of what one should be able to find. Their sense of possibility had shifted. Accordingly, they expressed frustration at the slow pace of the development, the underappreciation, as well as the ignorance of all the skill and labor that made efforts like Gene Ontology necessary and possible. One recurrent frustration was that research communities de facto acknowledged the need for knowledge bases as they constructed in-house depositories “typically as work package one in their projects”—just like the team in Trondheim had done annotating gene regulation information of the 500 genes they had printed on the array. By 2010, biologists lived in a world where they more or less took the need for knowledge bases for granted. The relation to an imagined future had shifted from the unfamiliar world of early post-genomics to one in which reliable tools could be expected to the point of frustration at their under-development.

Yet in 2010 things remained unsettled. Few researchers with significant resources were taking quality issues seriously enough to undertake the work of addressing them. By default, this left everyone in a position where they had to invent their own standards, collecting information for in-house use while not submitting it systematically to established public knowledge repositories like Gene Ontology. At the same time, by 2010 digital knowledge management was being turned into a research field. Researchers like those at Trondheim could now collaborate with professional curators in developing guidelines for the annotation of gene regulation, guidelines that would allow researchers to better fit the existing standards developed by actors like Gene Ontology, while simultaneously experimenting on extracting relevant information for such annotations through computer-assisted text mining.

Over the years, the imagined future had begun to be normalized to the point of being taken for granted—*despite the fact that it did not yet exist*. Daily activities—from writing grants, to animating research programs, organizing experiments, designing software, and hiring post-docs—began to be keyed to that imagined future. Once that happened, a wide range of additional future-oriented activities could be set into motion (e.g. the further refinement and elaboration of new ontologies, the organization of new initiatives and funding streams, the invention of new modes of expertise). The vision of a future driven by knowledge bases, in other words, began to be taken as not only scientifically desirable, but natural and thereby reasonable. In this situation the technology, talent, and resources needed to actualize a future where the digital object played a central role in the organizing, use, and even automation of reasoning with biological knowledge *has come to be treated as familiar*. Computers have long since been normalized in the lab, as in every area of our lives. Databases are not only commonplace but a quotidian part of the construction and execution of experimental practice. Organizations—e.g. the Gene Ontology Consortium (GO) or the European Bioinformatics Institute (EBI) —now undertake the immense curatorial labor needed to assure that the data put in databases is of sufficiently high quality.

For people like the curators and ontologists at EBI or GO, the work yet to be done remained painfully clear—the quality of data, the need for published work to take into account the requirements of data-management, the tendency of researchers to use their own data standards and even their own proprietary databases, and so on. But this work is taken to be self-evident in the sense that no one needs to think too much about whether it is the right thing to do. After all, biologists are already accustomed to sorting through massive amounts of data, conducting their work in view of a future in which computer-powered systems-level research has long become the cultural commonsense, even if it remains a long way off in practice.

Against this background, by 2017^5^ the GRECO initiative had gained enough momentum to gather key international experts in the field of gene regulation to set in motion extensive collaborative work. That work would aim to clarify and describe the scientific and technical standards by way of which state of the art understanding of gene regulation should be stored and shared ensuring interoperability across knowledge bases. By this point they had come to look at the present from a future point of view. This wasn’t simply a matter of planning or aspiration. It was rather, a way of contemporary challenges as obstacles for the realization of the imagined future. The longer-term transformation of knowledge infrastructures brought about by the inter-articulation of biological research and digital machines began, effectively, to be covered over. It began to be covered over in the sense that the key elements of the imagined future began to be taken for granted as the *simple extrapolation of infrastructural capacities already in place*.

Today, efforts to build knowledge bases as well as their omnipresent use are frequently cast as the natural next step for science. A white paper from the international society for biocuration is exemplary in this regard: “From the time when Henry Oldenburg published the first scientific journal in 1665 (Proceedings of the Royal Society) to the founding of the United States National Library of Medicine in 1879 to the present, there has been a sustained drive to improve how researchers can record and discover what is known” (ISB 2018, p.1). Researchers rely and build on existing knowledge, but the biologists’ infrastructures of knowledge management have not kept pace with its own fields “ever-faster rates” of generating data. Collecting information has become a demand of the day, one which leaves the individual’s understanding of the biological systems in question at risk of the very broad but superficial grasp of the biological situation that the Trondheim team first experienced in the late 1990s. The presumption of inevitability and naturalness generates an imperative, even where that imperative has not yet generated scientifically robust results. It is the expectation that those results will one day be robust that keeps the machinery moving.

In the midst of this, a wrinkle has gone unaddressed, though informally it is frequently brought up by the scientists involved. The wrinkle is this: what should we make, biologically, of the fact that research practices are expected to be adjusted to the requirements of the computer and the knowledge base, and not necessarily the other way around. Indeed, accommodating the demands for compliance and standardization in the name of interoperability has come to be taken as a given by those driving the creation of knowledge bases, almost to the point where the potential scientific limitations are no longer mentioned. One may get the impression that there is basically nothing new with knowledge bases. Digital knowledge repositories are a natural extension of the past—a publication for the twenty-first century. The work of monitoring current state of the art, what is currently known and demonstrated to be true, is something scientist have always done as part of their daily routine. The only difference is that prior knowledge is now expected to be collected, systemized and presented for the individual biologist by a digital platform that promises to do this better than individual researchers or research groups are able to do themselves. To quote the bio-curators’ whitepaper again: “The time wasted by individual researchers discovering information, collecting it, manually verifying it, and integrating it in a piecemeal fashion – all impede scientific advancement. For researchers, the work of biocurators ensures they can easily find extensive and interlinked information at well documented, stable resources. It means they can access this information through multiple channels by browsing websites, downloading it from repositories, or retrieving it dynamically via web services. It likewise means the information will be as accurate and reliable as possible” (ISB 2018, p.5).

Statements like this, we suggest, can be understood as an expression of how many in biology today have come to see the demands of research from a future digitalised point of view, a point of view taken to be familiar. In view of that familiarity, the work of building knowledge bases is taken up in a mode of confidence, where we can rest assured the traditional measures of scientific quality—if not yet with us—will one day be restored.

Importantly, once the imagined future is normalized and rendered familiar, responsibility for actualizing that future can be cast as a matter of *vocational necessity*. Members of the scientific community might disagree on specifics—not least the ongoing challenges to openly sharing data, given the politics of career development, publications, and intellectual property. Likewise, members could disagree on how far biology could be pushed by and through digital technologies—will artificial intelligence be adequate to biological experimentation if it can only find patterns but cannot provide causal explanations? The fact that the future of biology requires a digital research infrastructure is however not open for debate. Indeed, even biology itself is treated by some as nothing but a massive data problem. Hence, being a good scientist, being a responsible member of the community, is easily cast as requiring a commitment to building, maintaining and grounding design and interpretation of own research in shared digital infrastructures such as knowledge bases.

### The politics of reassurance

In such a situation, introducing knowledge bases may not be experienced as part of a significant shift of epistemic regimes, but rather as a natural extension of previous ones. It can be assumed that there is really nothing new, scientifically speaking, with knowledge bases. They are, so to speak, only a different form of a library. Once the unknown future has been domesticated, building knowledge bases no longer needs to be considered a research task. It becomes rather a question of motivation and management: one basically knows what work to be done as the quality standards are not basically new—one simply just needs to get to it and get the work done.

Once a vision of a future worth building becomes familiar, reasonable, and thus vocationally necessary, it becomes easier to think that the best way of achieving that future is through the assertion of institutional *reassurance* rather than the taking of scientific *risk*.

Take for example the efforts of an organization called ELIXIR. Headquartered at the Wellcome Genome Campus in the UK, ELIXIR is an “intergovernmental organisation that brings together life science resources from across Europe. These resources include databases, software tools, training materials, cloud storage and supercomputers.” ELIXIR’s goal is nothing less than “to coordinate these resources so that they form a single infrastructure” (ELIXIR home page). The goal is audacious. Literally thousands of databases and dozens of software tools are currently being used by researchers in Europe and elsewhere. The key word is *coordinate*. It suggests that a main blockage to the envisioned biological future is organizational and institutional management, and the adjustment of experimental practices therein. They may not, in fact, be wrong. But it is taken to follow that an appropriate means of getting scientists on board is through a reassurance of the preservation of the order of the past – someone needs to take on a role of coordinating efforts.

A major component of ELIXIR’s activities is the development of what they call Core Data Resources. The Core Data Resources, as imagined, will consist of “a set of European data resources of fundamental importance to the wider life-science community and the long-term preservation of biological data” (Durinx et al. 2017, p.1). Existing data tools can qualify to count as “Core Resources” to the extent that they meet certain criteria of quality and reliability as set and certified by ELIXIR. ELIXIR will reassure the broader research community by making sure that Core Resources have ongoing Governmental backing from the host country, sufficient to ensure long time preservation, maintenance, commitment and relevance over time. Such preservation and maintenance is resource intensive, requiring not only that content will be updated and corrected, but new structures be built to comply with changing norms of data management brought about by new theoretical developments.

Criteria for core recourses draw on well-established indicators for scientific reliability, they should for instance be well known and taken in use by key stakeholders, like relevant research communities, journals and funders. The politics of reassurance can likewise be seen in ways in which ELIXIR will use its institutional position to ensure and enforce: the *provenance* of data (data must be traceable back to a published text, to the evidence of scientific credibility therein); *evidence* (the way truth claims are established, and thus the reliability or trustworthiness of the claims); c*onfidence* (a measure that translates confidence into a number); *interoperability* (the assurance that digital objects can move and connect to other digital objects in other databases by way of standardization); *coverage* (the question of whether and to what extent a subfield is accounted for); and *maintenance* (the flexibility to adjust to new empirical findings.

The standards for core resources aim to establish high degrees of confidence in the quality of knowledge bases with reference to recognizable standards we are used to. One need not be a biologist to see the vital—but subtle—role of reassurance in stabilizing a vision of the future and working toward its realization here. How will data be made traceable? What will it mean for credibility to be based not just on the reputation of the journal publishing the work, but also on issues like evidence, maintenance, and interoperability? Who will determine whether a subfield is being taken care of? The scientific challenges entailed in each of these are significant but considered to be in reach seen from the domesticated future point of view. Hence the need for an organization, housed in an institution with the highest credibility, connected to other governmental institutions on the one side and the best science and engineering on the other. It may be difficult to achieve such level of assurance, but if anyone can do it ELIXIR can. Institutional reassurance replaces scientific risk.

The imagined future of digital infrastructures feels familiar. From that sense of familiarity the imagined future is taken to be an extension of the known past—despite the fact that scientific questions remain about whether this course of action will prove to be worth the effort. The reliability of these resources and thus their enduring credibility may also entail a willingness to take risks in relation to a series of scientific challenges that bear on matters of scientific quality. For example, will resource managers be able to ensure biological *relevance*—will the knowledge bases be biologically adequate, that is, adequate to the task of capturing and carrying out biological experimentation in a given subfield? Will it be scientifically *preferable*—will the hard work of complying with data standards and confidence measures pay back in increased quality for the working biologist? And will the metadata standards required for the computer, the database, and the curation of experimental results provide information that is rich enough for biologists to assess the relation between their own experimental systems and those of others? Will efforts to build and assure common quality standards, and common resources flatten the idiosyncratic richness of the multiplicity of recourses being created? Still more fundamental questions remain, such as to the question of the extent to which living organisms, their behaviour, and observations in their native and experimental contexts can be successfully captured by databases that have become standardized and interoperable in ways that may conceal fundamental differences between organisms, their contexts and their behaviours. These are scientific questions, and so bear directly on the implicit risks one is taking in moving toward a future in which a new set of tools, standards, modes of expertise and institutional forms—a new infrastructure—begins to triumph over the old.

## Part III: Between trust and confidence: ethical work and ethical modalities

The construction of knowledge bases has now become a well-established practice, and a mode of confidence may by this point be warranted. However, given the radical nature of the way knowledge bases destabilize knowledge infrastructures centered around natural language text (the paper), it is reasonable to ask whether the issue of trust has been foreclosed too early. We do not claim that the rise of post-genomics is the only reason why a re-engineering of the paper-centric knowledge infrastructures is called for—but that it has been a key factor in digitalisation of biological practice. Two decades ago, the researchers in our story of the microarray found themselves in a situation where breakdowns of the biologist’s lifeworld of the paper centric knowledge infrastructure was acutely felt. In choosing to pursue the microarray technology platform they turned their laboratory into an unfamiliar and unstable experimental system. This unfamiliar situation called for a trust mode, relatively radical measures had to be taken, and one could only hope that the microarray platform would prove itself to be a robust and reliable platform. The loss of a certain scientific resolution was obvious to everyone. Thousands of genes in each microarray experiment were observed at the cost of significant measurement noise and words pulled from abstracts functioned as a substitute for biological knowledge familiarity in interpreting these measurements.

But nevertheless it was a risk they were willing to take as microarrays carried a significant promise: researchers would be able to ask and answer a new class of questions. They were of course not alone in taking this step, microarrays constituted a key platform technology of post-genomics, one of many that would open up the possibility of system-wide studies.

We do not worry about risk taking, but about risk denial. The scientist’s inclination in situations like this is typically to embrace risk taking. Even though most researchers in the late 1990s, and even in the first decades of the new millennium preferred to stick to the old comfortable familiar research systems in lieu of greater dependency on computation, a substantial and increasing proportion of researchers chose to adopt system-wide experimental and knowledge management strategies. The inclination of such risk takers is to actively and continuously challengethe familiar and take on the hardships of restoring familiarity if it means opening potentially new lines of inquiry. As Isabella Stengers has argued this *should* even be the inclination of true scientists. Any scientific endeavor worthy of the name cannot avoid taking a certain risk. Science is defined precisely as a risky work of seeking to realize unknown futures (Stengers 1997; see also Bruno Latour’s introduction to Stenger). Any research task then, must move through a state of uncertainty, in which potential losses have to be risked, to a situation of restored familiarity in which the skills needed to act can yet again be treated as a normal feature of everyday life. But until and unless the unfamiliarity introduced by uncertainty is resolved, a *mode of trust would remain appropriate*, perhaps even necessary.

At stake here is the scientific virtue of risk-awareness or risk-perception. The virtue of risk-awareness has both epistemic and moral dimensions. Following Stengers, we think science needs to be risky because it explores the uncertain and thereby puts the status quo to the test: you can never be sure that you will get what you want. To cite another philosopher: it is the Popperian requirement to constantly put one’s theories at risk of being destabilized. But as Stengers notes, it is not only theories that are (and should be) put at risk of destabilization. It is also the world itself that should be put at risk, that is, the material and social conditions that give direction to and make scientific work possible. It is a world filled with actors who think, benefit, challenge, win, lose, dominate, recede, and discover. Science needs to be able to stand the trial of scientific and moral scrutiny.

This is where we find ourselves on the horns of a dilemma. The situation at hand is becoming relatively more robust. The predominant sense of confidence among the advocates of digitization may not be unwarranted. But that confidence is too often unalloyed and so obscures the places where risks might yet be in play and thus where work might yet be taken up—at least some of the time—in a mode of trust. It is certainly tricky to claim that we might be in a situation of epistemic risk even though most scientists involved do not experience it as such—or at least they do not talk about it in this way. This is why we have told the story of our research group’s work on building knowledge bases. The story is meant to show how the work of building knowledge bases, discussed in a particular case, includes risks by drawing attention to the way in which the work both introduces a significant destabilization, but also sets in motion a presumption of ultimate success, which is to say it domesticates the very future it imagines. The troubling scenario, as we see it, is that the presumption of success has come to dominate in a way that has effectively silenced the question of trust, a silencing which is scientifically and thereby also ethically troubling. In order to clarify what is at stake here we need to return to Luhmann’s key notion of familiarity.

### Disentangling the familiar and the unfamiliar

In the introduction we proposed that Luhmann’s distinction between trust and confidence can be thought of as two different modalities one can take in response to uncertainty and risk. Along the way we have also mentioned, but have not yet given careful attention to, a second distinction, one which can be thought of as analytically prior to the first. This second distinction is the familiar and unfamiliar. As can be surmised from the foregoing, the question of the relation between the familiar and the unfamiliar is central to the question of trust as we want to raise it in this paper.

Familiarity in Luhmann’s work refers to a state of being in the world—the “life-world” of phenomenology. A characteristic of one’s familiar lifeworld is that the conditions of skilful action (i.e. knowing what to do) are well understood. A lifeworld is one in which we have an expectation of what normally happens and what to do when it does. In order to live easily in the world, we learn to ignore a good deal of the risks that surround us. They have grown familiar. It is the case, of course, that many things in our lives, no matter how routine they have become, may not work out as expected. And yet, as Luhmann reminds us, we cannot constantly question the basic social mechanisms by way of which, collectively, we have come to rely on a course of action—despite the fact that things might not always work out (e.g. the risks inherent in driving a car, talking to strangers, turning on the stove to cook, and so on).

Familiar situations can thus be thought of as situations that allow one to proceed in a mode of confidence. The question for Luhmann is how to proceed when we encounter the unfamiliar? We must, he says, switch to a mode of trust in situations where the normal course of expectations can no longer be taken for granted. The task and challenge, for Luhmann, is ultimately to reestablish reliable routines that allow us to domesticate risks in a regularized fashion, thereby shifting back to a situation of restored familiarity in which the skills needed to navigate our lifeworld can yet again be treated as a normal feature of everyday life.

It is in light of this distinction between the familiar and the unfamiliar that we elaborated our idea of the domesticated future: to the extent that the future is uncertain (we do not know whether we will realize our plans) it would seem to call for a mode of trust. And yet, because the unknown future is actually experienced in the present as familiar—as an elaboration of what is expected—it seems to invite us to take up the work of building that imagined future in a mode of confidence. This tangle of the unknown yet familiar, we have suggested, has the effect of silencing the question of trust. That silencing, we want to propose in this last section, should be understood not only as a technical risk, but an ethical one: one in which we put at risk the very scientific goods that we hope for.

Luhman’s distinction, and the question of modalities it raises, helps us bring the ethical dimension to light. Familiarity in Luhmann’s sense shares important resonances with a term we have already introduced: Marcel Mauss’ moral habitus—our collective everyday sense of how the world works, and thus how to inhabit it in a way that seems right and good. In a stable situation, as anthropologist Jarret Zigon has explained, our moral habitus is sustained not primarily by adherence to “codes, or obligatory rule-following or conscious reflection on a problem or dilemma”. Our moral habitus, rather, is simply done. It is rarely even noticed when it is performed. It is familiar. It consists of, “one’s already cultivated everyday way of being in the world. It is because all persons are able to embody their morality in this unreflective and unreflexive way that most persons most of the time are able to act in ways that are, for the most part, acceptable to others in their social world seemingly naturally.” (Zigon 2010, p. 8).

But under circumstances where the stability and familiarity of existing research practices can no longer be taken for granted—for example, when the corpus of published papers became a problem for systems biologists—something crucial changes at the level of the moral habitus. Where a more or less taken for granted way of acting once held sway, a loss of familiarity is often experienced as breakdown or loss of sufficiency at a moral level. This may not happen all at once, and such a breakdown may never be total. It may begin only as a frustration or discomfort. But when existing research practices encounter the limits of the status quo, as in the story of the microarray, and disrupt a sense of familiarity, one would expect the moral habitus to be thrown into (partial) doubt, revisited, and taken up in a new, more conscious manner. At this point, to borrow a distinction from Zigon, the everyday experience of *moral habitus* switches over into a more deliberate *ethical work*, one in which a series of questions must be asked about how best to move forward—how best to establish the conditions needed to repair or otherwise restore a sense of moral habitus. Questions must be asked about ethical modalities, about whether and how to restore familiarity, thus warranting confidence, or whether the situation calls for the reflective and reflexive modality of trust.

In the case of knowledge infrastructures, a breakdown in familiarity might show itself in questions like: are the tools being developed flattening out scientific subtleties in a way that may, in the end, ultimately prove too costly at the level of our ability to understand and engineer living things? What might the life science become if it makes itself entirely dependent on computer-operations for interpreting biological observations? What world is about to be created and who gets to decide? In these cases, when the moral habitus of the research community becomes a problem, and to the extent that *this is found to be true*, the habitus itself, to quote the philosopher Michel Foucault, “is made to enter into the play of true and false” (D’Arcy 2004). It can thereby be tested, embraced, eschewed or renewed.

In the current situation, we fear that the shift in moral habitus brought about by the introduction of knowledge bases is not being experienced by the community as a problem and thus as a site of ethical reflection—even though it is possible that it should be given the extent to which it has destabilized knowledge practices and the infrastructures they rely on. The story of the building and appropriating of knowledge bases suggests that the dynamics of domestication may be muting scientific risks. Today, the digital lifeworld feels familiar. Because of this, the possible breakdown of moral habitus, one constantly at play in Stenger’s risky science, may simultaneously be suppressed as well. On the other hand, it may be that a mode of confidence prevails because the digital has put the question of risk in this domain to rest. It is an open question. Given the way ordinary research practice tends to suppress epistemic and moral reflection, we need to find ways to test whether a given situation calls for trust or confidence.

## Conclusion: toward a double-modality: un-silencing trust

At the heart of our story is the now-widespread sense that paper (i.e. natural language)- centric knowledge infrastructures can no longer adequately support life science research. As our story tries to show, existing knowledge infrastructures are experienced as inadequate in light of the questions researchers *find worth asking and the future answers they hope to generate* in the post genomic era. Knowledge bases, just as microarrays and other post-genomic technologies before them, have come to embody a vision of a future worth building. They are opening a door for new questions to be asked and new discoveries made. Without a better means of sharing, comparing, analyzing and modeling the massive new data post-genomics produced, the changes they introduced could not readily be stabilized. That restabilization could only begin to become possible when new scientific and technical practices began to develop in view of an imagined desired possible future. A future, that is, of knowledge bases.

Digital biological knowledge bases are built to support researchers in the life sciences. This requires an imagination of what future scientific world should be hoped for, and how machines must be built in order to make such a future possible. Knowledge bases, in short, are also a response to which biologists are imagining a different possible future, one they believe is *worth investing in*. Epistemic risks may be acknowledged in this situation, but they are nevertheless seen to be risks worth taking, considering what may be gained. Over time, however, as this paper suggests, the question of trust may come to be ignored as scientists get on with the daily nitty gritty work of building the ‘machines for making’ the desired future. Once digital infrastructures grow familiar, *the future imaginaries attached to them are backgrounded as well*. The hoped-for visions embodied in the machineries get normalized as a natural extension of where we’ve come from and where we’re going.

It’s that normalization—domestication—that we seek to trouble. Consider again the problem of the paper. Scientists are trained to handle scientific papers as a basic vehicle of scientific knowledge. They have, over time, carefully designed infrastructures to preserve, store and mobilise scientific knowledge by means of developing standardized paper formats, a culture of peer review processes, edited journals and library services. This infrastructure, despite its limitations and imperfections, has historically served the scientific community well. It is an integral part of scientific training to learn how to write, read, critically scrutinize and carefully weigh the scientific validity, find and refer to other scientists’ published findings. Scientists have thereby internalized ways of accessing knowledge in their field and ways of taking part in ongoing deliberations over what knowledge is reliable, trustworthy, and significant—and what is not.

Knowledge bases risk a fundamental reworking of this paper-centric infrastructure. The reasons are at once scientific and moral: for all its richness and subtlety the paper was not able to support genome-wide analyses, such as those carried out using microarray technology, to say nothing of the flood of data that is now produced on a daily basis in labs around the world. The construction and use of knowledge bases provides one possible way forward to mobilize computer-assisted interpretation, at scale, of the knowledge expressed in natural language texts. It has an evident downside. Compared to the scientific rigor scientists have grown used to in the reading and writing of papers, representing scientific knowledge in knowledge bases still has a way to go. Yet, at the same time, these digital infrastructures make possible a range of knowledge-coverage—a breadth of interrelationships that can be made operable through digital platforms—that simply cannot be seen, let alone made useful, at the level of detailed nuance older modalities might have allowed. As such, while it is reasonable to assume that knowledge bases still represent a risky epistemic move to the digital object as a basic vehicle for scientific knowledge, it is not yet clear whether and to what extent such a risk is worth taking.

But the problem is more than a matter of scientific recalibration. It is a moral vision for a different future, one which carries with it a reworking of the moral habitus. The work of reengineering knowledge infrastructures requires reimagining which questions can be asked and thus which questions are worth asking and who gets to decide. It requires a reorientation to the future of the enabling infrastructure itself—a reorientation which induces shifts of aspiration, commitment and experimentation. The aim of the work of reengineering is reliability which translates into building *robust* knowledge infrastructures. Success is often determined pragmatically: by the ability of the knowledge infrastructure to help generate and circulate questions and possible answers the community aims for. Yet in actual practice then, the work of reengineering knowledge infrastructures is less obvious. It involves a re-calibration of the *ethos* of the research community itself. The work of reengineering is not only technical it is simultaneously also moral work: it is a techno-moral work.

Ethos, as is worth remembering, refers in its everyday English usage to the “habitual character and disposition” of a place or community—its “moral character; habit, custom; an accustomed place”( Online etymology dictionary). The ethos is the moral habitus that is socially attained over a lifetime. Indeed, the word “moral” is etymologically linked to the work ethos: it refers to the *manner* in which something gets properly done, as seen by the standards of the community. The habitual, and therefore familiar, manner of living that comes to define the ethos of a community or culture and the place of an individual therein.

The work of building knowledge bases, as our story aims to show, unsettles or destabilises familiar practices of knowledge production, management, and consumption. And yet, the domestication of the future potentially conceals that destabilization rather than addressing it directly in a manner that would allow researchers to weigh the potential risks involved in their work. That covering over, or silencing, brings with it important consequences for not only the development of new techniques and technologies, but for the moral life of the research community as well. The character of the communities’ work is affected: what their work aims at, who and what institutions they interact with, what is taken to be worth doing and how. If the risks involved in the work are covered over, if the problem of trust is silenced, the biological community may risk unwarranted epistemic blockages and unexamined breakdowns of moral habitus. Equally, they may miss the opportunity to rebuild that habitus and overcome those breakdowns.

The distinction between working in a mode of confidence and a mode of trust thus makes a difference. To the extent that the construction of knowledge bases calls for trust, researchers might be expected to embrace and operate in a reflexive work mode, remaining open to possible alternatives and attentive to reasons for resistance and non-compliance. If it warrants a mode of confidence, by contrast, the expectation is that the hoped-for future does not entail risks, in the sense of remaining open to doubts about the direction to take, even if it may not be realized. Rather, it entails a coupling of normalization and hard work realized through commitment and collaboration. That commitment and collaboration may not come to fruition. Knowledge bases may not be constructed or used in a sufficiently reliable manner. In this sense, the risk is still there, but it is a sort of risk one might describe as a *calculated* risk. The work of achieving the desired outcome can, in this case, be considered as a matter of routine: of establishing the processes, division of labor, and resources needed to bring things about. In such a mode, alternative courses of action need not really be considered. Indeed, such consideration may be counted as an opportunity cost—delays that hurt more than they promise to help. In such a mode, one is first and foremost committed to getting on with the work that needs to be done. While resistance may be understandable it is not reasonable given that one works in a mode of confidence – building them is a matter of vocational necessity. A trust mode in contrast, is marked by caution and an uncertainty regarding the prospects of building a robust knowledge infrastructure that may withstand scientific, moral and political scrutiny.

The mode of trust is a response to a risk that may not go away—a risk that cannot be domesticated, in the sense that one never fully feels at home with it, even if one learns to live with it. It may nevertheless very well be that the now decades-long effort to translate and transform natural language representations into the digitization of knowledge in biology has grown so familiar that, despite persistent risks, it now appears to be primarily a question of confidence. It has, in this sense, become domesticated. Trust is silenced—but it shouldn’t be. Given the uneasy relation of risk and familiarity at the intersection of computer tools and biological science the question remains an open one. What remains then, is to take up the challenge—one we have not met sufficiently in our own work—of undoing the silencing or at least of discovering how best to *test* whether trust may yet be required.

In this paper, we have described how the knowledge infrastructures that support biology are shifting, and that one source of that shift is the construction of knowledge bases. Such shifts de-stabilize not only technical infrastructures, but also the ethos, which is to say moral habitus, of the people and institutions that rely on those infrastructures. The story of the microarray is a story of how a need for new knowledge infrastructures manifested itself in a research group developing a post-genomic technological platform. To release the imagined and desired potential of microarrays some form of digital knowledge bases had to be brought into the world. The introduction of knowledge bases constitutes part of a significant transformation of practices currently taking place in the life sciences.

Whether a mode of confidence or a mode of trust is warranted cannot, in our view, be answered by appealing to whether the situation at hand feels like a familiar one. Familiarity, as we have shown, can be established through a mechanism of domestication of the future. The question of trust must, in our view, be openly discussed. To open up such a discussion however remains challenging. A world dominated by computers, after all, may be considered risky in an existential sense, but usually it is taken for granted scientifically. The question of trust cannot be productively asked in the current situation because of the ways in which digital infrastructures have grown familiar. Rather than a strict alternative, we suspect the question of trust or confidence is one of ratios: when and under what conditions must work conducted under conditions of familiarity be interrupted by a deliberate return to the question of trust—by a deliberate reminder of the permanent possibility of, and need for, the unfamiliar in a space of scientific reason. Once the question of trust is un-silenced, if indeed it can be, then deciphering these ratios will be, for us, the work that lies ahead.

## Footnotes

1 See, for example, the Gene Ontology Consortium https://geneontology.org/, the European Bioinformatics Institute https://www.ebi.ac.uk/, and the Gene Regulation Consortium https://thegreco.org/.

2 GRECO (https://www.thegreco.org/) is a bottom up initiative taken by researchers engaged in gene regulation research. The initiative has been supported at the European level as a COST Action (GREEKC project, https://www.greekc.org/).

3 A knowledge resource for a domain like gene regulation does not need to be stored in one facility or depository, but may also be distributed among many facilities.

4 Westerhoff and Palsson (2004) describe a parallel development of HTP technology and mathematical frameworks for data analysis.

5 The first workshop of GREEKC projectwhere held in April 2017 (https://greekc.org/).
